# Determination of digestible indispensable amino acid score for salmon hydrolysate proteins

**DOI:** 10.1002/jsfa.70518

**Published:** 2026-02-19

**Authors:** Natalia S Fanelli, Juliana CFR Martins, Hans H Stein

**Affiliations:** ^1^ Department of Animal Sciences University of Illinois Urbana IL USA

**Keywords:** amino acids, digestibility, digestible indispensable amino acid score, fish, protein quality

## Abstract

**BACKGROUND:**

Salmon products are excellent foods that contain indispensable nutrients including fatty acids and amino acids (AA), but during processing, salmon co‐products that cannot be used for the primary purpose are also generated. Examples of such co‐products include salmon protein hydrolysate concentrate (SPHC) and salmon protein hydrolysate isolate (SPHI), but there is no information about the protein quality of these co‐products. Therefore, the objective of this experiment was to use the digestible indispensable amino acid score (DIAAS) method to test the hypothesis that the protein in SPHC and two sources of SPHI (SPHI1 and SPHI2) can supplement lower‐quality proteins.

**RESULTS:**

For children from 6 months to 3 years old and individuals older than 3 years, SPHC had greater (*P* < 0.05) DIAAS than SPHI1 and SPHI2, and SPHI2 had greater (*P* < 0.05) DIAAS than SPHI1. For children from 6 months to 3 years, leucine was the first limiting AA in SPHC, and tryptophan was the first limiting AA in SPHI1 and SPHI2. For individuals older than 3 years, there was no limiting AA (DIAAS ≥ 100) for SPHC, but for SPHI1 and SPHI2 leucine was the first limiting AA.

**CONCLUSION:**

All sources of salmon protein hydrolysates had excellent AA digestibility, and SPHC can be used to compensate for lower protein quality in other ingredients to produce a balanced diet that meets requirements for all indispensable AA for individuals older than 3 years. © 2026 The Author(s). *Journal of the Science of Food and Agriculture* published by John Wiley & Sons Ltd on behalf of Society of Chemical Industry.

## INTRODUCTION

Protein is an essential nutrient required by the human body due to requirements for indispensable amino acids (AA) that are needed for protein synthesis and other functions.^1^ To ensure the correct balance among AA, protein quality in human foods needs to be determined. The digestible indispensable amino acid score (DIAAS) is the preferred method to evaluate protein quality in foods because it is based on determination of AA absorption before the end of the small intestine (the ileum), which is more accurate than determining total tract digestibility of AA due to microbial fermentation in the hindgut.^2^ Because of the difficulty of determining ileal digestibility of AA in humans, the pig is often used as the preferred model,^2^ and values for AA digestibility in pigs are in close agreement with values obtained in humans.^3^

Salmon provides essential nutrients to diets, including indispensable AA, omega‐3 fatty acids, vitamins, and minerals, which make salmon an excellent dietary choice, especially for young children and people with high AA requirements.^4^ Processing of salmon to generate salmon fillets or other products generates co‐products, which can be used to produce salmon hydrolysate proteins. Hydrolysate proteins are considered superior to intact proteins because they are pre‐digested by enzymes, resulting in smaller peptides that presumably have improved digestibility of AA.^4^, ^5^ Indeed, greater digestibility of AA has been demonstrated for salmon, squid, and shrimp protein hydrolysates compared with intact fish meals when fed to pigs, dogs, or roosters.^6^, ^7^, ^8^, ^9^, ^10^

Protein quality of porcine and bovine protein hydrolysates has been reported,^11^ but no data demonstrating protein quality in salmon protein hydrolysates for humans are available, which restricts their use in meals that are prepared with the objective of balancing indispensable AA. Therefore, the objective of this experiment was to determine DIAAS in three novel sources of salmon protein hydrolysates and to test the hypothesis that these proteins can be used to supplement lower‐quality proteins and create meals that are balanced in AA.

## MATERIALS AND METHODS

The protocol was reviewed and approved by the Institutional Animal Care and Use Committee at the University of Illinois prior to initiation of the experiment. Female pigs that were the offspring of Line 800 boars mated to Camborough females (Pig Improvement Company, Hendersonville, TN, USA) were used.

### Ingredients and experimental diets

Three novel salmon protein hydrolysates, including one source of salmon protein hydrolysate concentrate (SPHC) and two sources of salmon protein hydrolysate isolates (SPHI1 and SPHI2), were produced by Biomega Group AS, Skageneset, Norway (Table 1). The SPHC was produced from raw food‐grade salmon materials, and included bone, offcuts, and viscera. The viscera went through autolysis (hydrolysis by endogenous proteases) before being heated to at least 85 °C and separated into three fractions: oil, water‐soluble, and water‐insoluble fractions. The oil was removed, whereas the other two fractions were mixed with the bones and non‐soluble fraction from the offcut line (leftover trimmings and scraps) before being dried in a disc‐drier and milled.

**Table 1:** Analyzed nutrient composition of ingredients (as‐fed basis)^a^

Item, %	SPHC	SPHI1	SPHI2
Dry matter	96.69	96.16	96.37
Crude protein^b^	68.63	92.75	91.95
Indispensable amino acids			
Arginine	4.10	5.28	6.63
Histidine	1.59	1.63	2.07
Isoleucine	2.83	2.21	2.92
Leucine	4.40	4.15	4.83
Lysine	4.76	5.33	7.08
Methionine	1.83	1.75	2.34
Phenylalanine	2.54	2.14	2.66
Threonine	2.82	2.81	3.66
Tryptophan	0.92	0.47	0.66
Valine	3.27	2.84	3.69
Dispensable amino acids			
Alanine	4.24	6.28	7.18
Aspartic acid	5.91	6.70	9.16
Cysteine	0.65	0.33	0.53
Glutamic acid	7.87	10.54	13.61
Glycine	6.15	11.32	13.10
Proline	3.46	5.48	6.79
Serine	2.43	2.83	3.53
Tyrosine	1.49	0.99	1.50
Total amino acids	61.26	73.08	91.94
Lysine:crude protein	6.94	5.75	7.70
Non‐protein nitrogen^c^	7.37	19.67	0.01

^a^ SPHC, salmon protein hydrolysate concentrate; SPHI1, salmon protein hydrolysate isolate 1; SPHI2, salmon protein hydrolysate isolate 2.

^b^ Crude protein for SPHC and SPHI1 was calculated as nitrogen × 6.25, but for SPHI2, crude protein was calculated as nitrogen × 5.60.

^c^ Non‐protein nitrogen was calculated as: crude protein − total amino acids.

The SPHI1 and SPHI2 were produced from raw food‐grade salmon materials (offcut without viscera) using a commercial food‐grade protease in a patented process (Biomega Group AS). After enzymatic hydrolysis, all fractions were heated to at least 85 °C before the water‐soluble content was separated from the oil and non‐soluble fractions by centrifugal force. In the production of SPHI1, the water‐soluble fraction was spray‐dried without prior filtration, but in the production of SPHI2 the water‐soluble fraction was ultrafiltered, and the permeate from the ultrafiltration was passed through a nano‐filter. The retentate from the nanofiltration was concentrated in an evaporator before spray‐drying.

Three diets were formulated by including each of the three salmon protein hydrolysates in one diet as the only source of crude protein (CP) and AA. A nitrogen‐free diet was also formulated and used to determine basal endogenous losses of CP and AA. Therefore, a total of four diets were used (Tables 2 and 3). Diet formulation was adjusted to obtain approximately 10% CP in all diets (dry matter (DM) basis) as recommended.^12^ All diets included vitamins and minerals to meet or exceed nutrient requirement estimates for swine,^9^ and 0.40% titanium dioxide was used as an indigestible marker. Samples of each ingredient and diet were collected at the time of diet mixing and used for chemical analysis.

**Table 2:** Ingredient composition of experimental diets (as‐fed basis)^a^

Ingredient (%)	SPHC	SPHI1	SPHI2	Nitrogen‐free
Experimental protein	14.00	10.80	10.80	—
Corn starch	66.25	68.00	67.50	78.10
Sucrose	10.00	10.00	10.00	10.00
Canola oil	5.00	5.00	5.00	5.00
Solka floc	3.00	3.00	3.00	3.00
Dicalcium phosphate	—	1.00	1.50	1.70
Ground limestone	0.25	0.80	0.50	0.40
Sodium chloride	0.40	0.40	0.40	0.40
Magnesium oxide	0.10	0.10	0.10	0.10
Potassium carbonate	0.10	—	0.30	0.40
Titanium dioxide	0.40	0.40	0.40	0.40
Vitamin–mineral premix^b^	0.50	0.50	0.50	0.50

^a^ All diets, except the nitrogen‐free diet, were formulated to contain approximately 10% crude protein (dry matter basis).

^b^ The vitamin–micromineral premix provided the following quantities of vitamins and microminerals per kilogram of complete diet: vitamin A as retinyl acetate, 11 136 IU; vitamin D_3_ as cholecalciferol, 2208 IU; vitamin E as dl‐α‐tocopheryl acetate, 66 IU; vitamin K as menadione dimethylprimidinol bisulfite, 1.42 mg; thiamine as thiamine mononitrate, 0.24 mg; riboflavin, 6.59 mg; pyridoxine as pyridoxine hydrochloride, 0.24 mg; vitamin B_12_, 0.03 mg; d‐pantothenic acid as d‐calcium pantothenate, 23.5 mg; niacin, 44.1 mg; folic acid, 1.59 mg; biotin, 0.44 mg; Cu, 20 mg as copper sulfate and copper chloride; Fe, 126 mg as ferrous sulfate; I, 1.26 mg as ethylenediamine dihydriodide; Mn, 60.2 mg as manganese sulfate; Se, 0.3 mg as sodium selenite and selenium yeast; and Zn, 125.1 mg as zinc sulfate.

Abbreviations: SPHC, salmon protein hydrolysate concentrate; SPHI1, salmon protein hydrolysate isolate 1; SPHI2, salmon protein hydrolysate isolate 2.

**Table 3:** Analyzed nutrient composition of experimental diets (as‐fed basis)

Item (%)	SPHC	SPHI1	SPHI2	Nitrogen‐free
Dry matter	92.63	92.26	91.98	91.40
Crude protein^a^	9.76	10.00	9.88	0.27
Indispensable amino acids				
Arginine	0.51	0.55	0.69	0.01
Histidine	0.23	0.19	0.24	0.01
Isoleucine	0.40	0.26	0.35	0.01
Leucine	0.63	0.49	0.58	0.03
Lysine	0.67	0.62	0.81	0.02
Methionine	0.25	0.21	0.25	0.01
Phenylalanine	0.36	0.24	0.31	0.02
Threonine	0.38	0.32	0.42	0.01
Tryptophan	0.11	0.05	0.07	0.02
Valine	0.46	0.33	0.43	0.02
Dispensable amino acids				
Alanine	0.60	0.73	0.84	0.02
Aspartic acid	0.84	0.78	1.07	0.02
Cysteine	0.08	0.04	0.06	0.01
Glutamic acid	1.17	1.29	1.63	0.04
Glycine	0.85	1.30	1.52	0.01
Proline	0.49	0.63	0.78	0.02
Serine	0.35	0.36	0.45	0.01
Tyrosine	0.18	0.10	0.14	0.01
Total amino acids	8.56	8.49	10.64	0.30

Abbreviations: SPHC, salmon protein hydrolysate concentrate; SPHI1, salmon protein hydrolysate isolate 1; SPHI2, salmon protein hydrolysate isolate 2.

^a^ Crude protein for the SPHC and SPHI1 diets was calculated as nitrogen × 6.25, but for SPHI2 diet crude protein was calculated as nitrogen × 5.60.

### Experimental design and digestibility experiment

Twelve growing gilts (average initial body weight: 85.8 ± 9.1 kg) with a T‐cannula in the distal ileum were randomly allotted to quadruplicated 3 × 2 Youden squares, which included three salmon protein diets and two 7‐day periods, for a total of eight observations per treatment. All pigs received a nitrogen‐free diet during the third period to determine basal endogenous AA losses, allowing each pig to serve as its own control for calculating standardized ileal digestibility (SID).^12^ The T‐cannulas were surgically installed in the pigs when they had an average initial body weight of 30 kg;^13^ the pigs had been used in two previous experiments before the current experiment. However, before being used in the present experiment, they were fed a common grower phase diet for 2 weeks.

Pigs were housed in individual pens (1.5 × 2.5 m) in an environmentally controlled room. Pens had smooth sides and partially slatted floors, and a feeder and a nipple drinker were installed in each pen. All pigs were fed their assigned diets in a daily amount equivalent to 8% of body weight^0.75^ calculated on a DM basis. The daily feed provisions were divided into two equal meals that were provided every day at 0700 and 1600 h.^12^ Water was available at all times and the amount of feed supplied each day was recorded. Pig weights were recorded at the end of each experimental period to calculate feed allowance for the following period.

The initial 5 days of each period were considered an adaptation period to the diet, and ileal digesta were collected for 9 h on days 6 and 7 according to standard procedures.^13^, ^14^ A plastic bag was attached to the cannula barrel, and digesta flowing into the bag were collected. Bags were removed whenever they were filled with digesta, or at least once every 30 min, and immediately frozen at −20 °C to prevent bacterial degradation of the AA in the digesta. At the conclusion of the experiment, pigs had an average final body weight of 92.4 ± 10.0 kg.

### Chemical analysis

At the conclusion of the experiment, ileal digesta samples were thawed, mixed within animal and diet, and a subsample was collected for chemical analysis. Ileal digesta samples were lyophilized (freeze‐dryer Gamma 1‐16 LSCplus, IMA Life, Christ, Osterode am Harz, Germany) and finely ground prior to chemical analysis. Samples of all ingredients, diets, and ileal digesta were analyzed for DM (AOAC Official Method 930.15),^15^ and AA were analyzed according to AOAC Official Method 982.30E (a, b, c)^15^ on an AA analyzer (model L8800, Hitachi High Technologies America, Inc., Pleasanton, CA, USA). Nitrogen was analyzed by combustion (AOAC Official Method 990.03)^15^ using a LECO FP628 nitrogen analyzer (LECO Corp., Saint Joseph, MI, USA). Crude protein in ingredients, diets, and ileal digesta was calculated as nitrogen × 6.25, except for SPHI2, for which a nitrogen conversion factor of 5.60 was used according to the manufacturer's specifications. Diets and ileal digesta samples were also analyzed for titanium.^16^

### Calculations

The apparent ileal digestibility (AID), basal endogenous losses, and SID of CP and all AA in each diet were calculated.^17^ Subsequently, the quantity (mg) of SID AA per gram of protein in each diet was calculated by multiplying the digestibility for each AA by the AA concentration in each protein. This quantity was then divided by the reference value for each indispensable AA to calculate the digestible indispensable AA reference ratio for each AA using the following equation:^2^

Digestible indispensable AA reference ratio = Digestible indispensable AA content in 1 g protein of food (mg) / mg of the same dietary indispensable AA in 1 g of reference protein.

The DIAAS values were calculated for children (from 6 months to 3 years old) and for older children, adolescents, and adults (individuals older than 3 years) using the following equation^2^:

DIAAS (%) = 100 × Lowest value of digestible indispensable AA reference ratio.

The DIAAS values for the salmon protein hydrolysates were calculated based on the CP content using the 6.25 nitrogen conversion factor as recommended.^2^ However, for SPHI2, the DIAAS values were also calculated using the 5.60 nitrogen factor to provide a more accurate value for this ingredient.

### Statistical analysis

Data were analyzed using the MIXED procedure of SAS (9.4 version, SAS Institute, Cary, NC, USA) using the pig as the experimental unit. Normality of residuals and homogeneity of variances were confirmed using the UNIVARIATE procedure of SAS. Brown and Forsythe's test was used to confirm variance homogeneity and, when this assumption was not met, data were transformed using the BOXCOX procedure, and assumptions were verified. Outliers were detected as observations that deviated from 1st or 3rd quartiles by ±3 times the interquartile range and removed from the treatments. The statistical model included diet as the fixed effect, whereas pig, square, and period were considered random effects. Treatment means were calculated using LSMeans and, if significant, means were separated using the PDIFF option in the MIXED procedure. Results were considered significant at *P* < 0.05.

## RESULTS

The AID of most AA was greater (*P* < 0.05) in SPHC compared with SPHI1 and SPHI2, with the exception that for the AID of methionine no difference was observed between SPHC and SPHI1, and for the AID of serine no difference was observed between SPHC and SPHI2 (Table 4). The AID of CP was greater (*P* < 0.05) for SPHC and SPHI1 than for SPHI2. The AID of arginine and glycine were greater (*P* < 0.05) for SPHI1 and SPHI2 compared with SPHC. No differences were observed for AID of lysine, alanine, and glutamic acid among the three salmon hydrolysate proteins.

**Table 4:** Apparent ileal digestibility of crude protein and amino acids in experimental ingredients^†^

Item (%)	SPHC	SPHI1	SPHI2	SEM	*P*‐value
Crude protein	73.3a	74.6a	68.5b	1.97	0.048
Indispensable amino acids					
Arginine	81.9b	87.2a	87.1a	1.65	0.009
Histidine	83.3a	79.7b	79.9b	0.87	0.005
Isoleucine	84.0a	77.1c	80.3b	0.93	0.001
Leucine	85.5a	81.1b	82.0b	0.71	0.001
Lysine	83.3	82.9	83.5	0.95	0.884
Methionine	88.3a	86.2ab	84.5b	0.87	0.013
Phenylalanine	84.2a	77.8c	80.5b	0.91	0.001
Threonine	76.1a	67.8c	72.0b	1.33	0.001
Tryptophan	83.6a	69.3b	72.4b	2.33	<0.001
Valine	83.0a	76.9b	79.3b	0.90	0.001
Dispensable amino acids					
Alanine	80.6	83.8	82.3	1.60	0.131
Aspartic acid	70.9a	60.6b	55.5b	3.00	0.005
Cysteine	56.1a	−7.4c	18.2b	3.86	<0.001
Glutamic acid	83.7	83.2	83.4	0.94	0.912
Glycine	70.5b	78.8a	76.2a	2.96	0.032
Serine	75.6a	72.0b	73.7ab	1.46	0.032
Tyrosine	79.9a	64.5c	72.0b	1.46	<0.001

*Note*: Means within a row lacking a common letter (a–c) differ (*P* < 0.05).

Abbreviations: SEM, standard error of the mean; SPHC, salmon protein hydrolysate concentrate; SPHI1, salmon protein hydrolysate isolate 1; SPHI2, salmon protein hydrolysate isolate 2.

^†^ Data are means of 8 observations per treatment, except for the SPHI1 that had 6 observations per treatment due to statistical outliers.

The SID of CP, histidine, methionine, and tryptophan were greater (*P* < 0.05) in SPHC and SPHI1 compared with SPHI2 (Table 5). The SID of threonine, cysteine, and serine were greater (*P* < 0.05) for SPHC than for SPHI1 and SPHI2, but for the SID of aspartic acid no difference was observed between SPHC and SPHI1. In addition, no differences among the three salmon hydrolysate proteins were observed for the SID of the remaining AA.

**Table 5:** Standardized ileal digestibility (SID) of crude protein and amino acids in experimental ingredients^†^

Item (%)	SPHC	SPHI1	SPHI2	SEM	*P*‐value
Crude protein	94.0a	95.0a	88.6b	2.48	0.032
Indispensable amino acids					
Arginine	99.0	102.8	99.4	2.29	0.100
Histidine	93.5a	92.4a	89.7b	0.86	0.007
Isoleucine	93.9	92.6	91.7	0.83	0.117
Leucine	94.8	93.6	92.5	0.71	0.061
Lysine	94.5	95.4	93.0	0.90	0.141
Methionine	92.8a	91.9a	89.3b	0.87	0.016
Phenylalanine	93.9	93.1	92.3	0.86	0.382
Threonine	92.1a	87.0b	86.6b	1.09	0.022
Tryptophan	93.1a	91.5a	87.9b	1.87	0.011
Valine	93.7	91.9	90.8	0.96	0.077
Dispensable amino acids					
Alanine	94.4	95.6	92.3	1.59	0.091
Aspartic acid	82.2a	73.4ab	64.6b	2.99	0.002
Cysteine	87.2a	56.7b	62.1b	3.43	<0.001
Glutamic acid	93.7	92.8	90.8	1.00	0.062
Glycine	93.2	93.0	88.7	2.86	0.168
Serine	92.7a	89.1b	87.1b	1.09	0.002
Tyrosine	94.0	91.7	91.2	1.50	0.361

*Note*: Means within a row lacking a common letter (a–c) differ (*P* < 0.05).

^†^ SID values were calculated by correcting values for basal ileal endogenous losses for each pig as its own control. However, average values of basal ileal endogenous losses (g kg^−1^ dry matter intake) were calculated as follows: CP 21.57, arginine 0.91, histidine 0.26, isoleucine 0.43, leucine 0.65, lysine 0.82, methionine 0.12, phenylalanine 0.38, threonine 0.66, tryptophan 0.12, valine 0.53, alanine 0.89, aspartic acid 1.05, cysteine 0.27, glutamic acid 1.29, serine 0.65, and tyrosine 0.28. Data are means of 8 observations per treatment, except for SPHI1, which had 6 observations per treatment due to statistical outliers.

Abbreviations: SEM, standard error of the mean; SPHC, salmon protein hydrolysate concentrate; SPHI1, salmon protein hydrolysate isolate 1; SPHI2, salmon protein hydrolysate isolate 2.

For children from 6 months to 3 years old and individuals older than 3 years, SPHC had greater (*P* < 0.05) DIAAS compared with SPHI1 and SPHI2, and SPHI2 had greater (*P* < 0.05) DIAAS than SPHI1 (Table 6). For children, leucine was the first limiting AA for SPHC and tryptophan was the first limiting AA for SPHI1 and SPHI2. For individuals older than 3 years, there was no limiting AA (DIAAS ≥100) for SPHC, but for SPHI1 and SPHI2 leucine was the first limiting AA.

**Table 6:** Reference ratios and digestible indispensable amino acid score (DIAAS) in salmon hydrolysate proteins^†^

Item	SPHC	SPHI1	SPHI2^‡^	SEM	*P*‐value
Child reference ratio^§^					
Histidine	1.08	0.81	0.90 [1.01]		
Isoleucine	1.21	0.69	0.82 [0.91]		
Leucine	0.92	0.63	0.66 [0.74]		
Lysine	1.15	0.96	1.13 [1.26]		
SAA	1.23	0.71	0.87 [0.97]		
AAA	1.06	0.60	0.72 [0.80]		
Threonine	1.22	0.85	1.00 [1.11]		
Tryptophan	1.47	0.55	0.66 [0.74]		
Valine	1.04	0.65	0.76 [0.85]		
DIAAS^*¶*^, (%)	92a (leucine)	55c (tryptophan)	66b [74] (tryptophan)	1.00	<0.001
Older child, adolescent, adult reference ratio^¥^					
Histidine	1.35	1.01	1.13 [1.26]		
Isoleucine	1.29	0.74	0.87 [0.97]		
Leucine	1.00	0.69	0.71 [0.80]		
Lysine	1.37	1.14	1.34 [1.49]		
SAA	1.44	0.83	1.02 [1.14]		
AAA	1.35	0.76	0.91 [1.01]		
Threonine	1.51	1.05	1.24 [1.38]		
Tryptophan	1.89	0.70	0.86 [0.96]		
Valine	1.12	0.70	0.82 [0.91]		
DIAAS^¶^, (%)	100a	69c (leucine)	71b [80] (leucine)	0.76	<0.001

*Note*: Means within a row lacking a common letter (a–c) differ (*P* < 0.05).

^†^ AAA, aromatic amino acids (phenylalanine + tyrosine); SAA, sulfur amino acids (methionine + cysteine); SEM, standard error of the mean; SPHC, salmon protein hydrolysate concentrate; SPHI1, salmon protein hydrolysate isolate 1; SPHI2, salmon protein hydrolysate isolate 2. First‐limiting AA is in parentheses.

^‡^ Values in brackets for SPHI2 represent values for DIAAS calculated using the 5.60 nitrogen conversion factor for CP in this ingredient.

^§^ The DIAAS were calculated using the recommended AA scoring pattern for a child (6 months to 3 years). The indispensable AA reference patterns are expressed as mg AA/g protein: Histidine, 20; Isoleucine, 32; Leucine, 66; Lysine, 57; Sulfur AA, 27; Aromatic AA, 52; Threonine, 31; Tryptophan, 8.5; Valine, 43.^2^

^¶^ DIAAS claims: < 75 = no protein quality claims; between 75 and – – 99 = ‘“good’” protein quality; ≥ 100 = ‘“excellent’” protein quality.^2^

^¥^ The DIAAS were calculated using the recommended AA scoring pattern for an older child, adolescent, and adult (older than 3 years). The indispensable AA reference patterns are expressed as mg AA/g protein: Histidine, 16; Isoleucine, 30; Leucine, 61; Lysine, 48; Sulfur AA, 23; Aromatic AA, 41; Threonine, 25; Tryptophan, 6.6; Valine, 40.^2^

## DISCUSSION

Salmon is a widely consumed food and includes several species that may be caught in the wild or produced in fisheries or aquaculture systems. Since the mid‐1990s, production of farmed salmon has surpassed that of wild‐caught salmon and now represents 80% of the global salmon supply.^18^ Salmon is primarily used to produce salmon fillets or steaks, but co‐products are also generated, although considered low‐value products. However, hydrolyzation of some of the co‐products including heads, bones, and skin may increase their values.^19^ The process of making salmon protein hydrolysates involves hydrolyzing the proteins into smaller peptides and AA, which enhances their nutritional and functional properties.^20^, ^21^ This process uses specific enzymes to hydrolyze the proteins at specific sites.^22^ The choice of temperature, enzymes, pH, and process duration are critical factors that influence the yield, molecular weight distribution, and bioactive properties of the resulting hydrolysates. After enzymatic hydrolysis, the mixture undergoes centrifugation or filtration to remove insoluble materials.^22^ The liquid hydrolysates can then be dried to obtain a powdered form that can be incorporated into food preparations. Therefore, protein hydrolysates may be used in food and beverage formulations, as well as dietary supplements. There is, however, a lack of knowledge about the protein quality of salmon hydrolysis co‐products for human consumption, and the current research was conducted to help fill this gap.

The AA composition of the salmon protein hydrolysates indicated that tryptophan and cysteine were the AA with lowest concentrations, which is in agreement with published data for these proteins.^23^ Nutrient composition analysis indicated that the intended concentrations of CP and AA were present in all diets. Pigs that received the diet with SPHC readily consumed their assigned meal, but pigs receiving diets containing SPHI1 or SPHI2 did not consume all of their daily allotted feed (average refusal SPHI1: 40%; SPHI2: 38%), which may be attributed to palatability issues because fish protein hydrolysates may have a bitter taste due to the presence of hydrophobic AA that are exposed during the hydrolysis process.^24^ However, the recovery of the indigestible marker was as expected and digestibility of AA was not believed to have been affected by the reduced feed intake of these proteins.

The SID of AA demonstrated that threonine was the indispensable AA with the lowest digestibility, which is because the greatest source of endogenous protein is mucin, which is high in threonine.^25^, ^26^ Values for digestibility of AA in the salmon proteins were also generally in agreement with published data for digestibility of AA in salmon protein hydrolysate, with cysteine having the lowest SID among all AA.^6^, ^23^, ^27^ The lysine:CP for all salmon proteins was between 5.7% and 7.7%, compared with 5.5% in a regular salmon protein hydrolysate, indicating that the drying methods used in the production of these proteins did not destroy lysine. The SID for most indispensable AA was not different among the three hydrolysates, but the observation that SID of histidine, methionine, and tryptophan were less in SPHI2 compared with SPHC and SPHI1 may be due to the addition of the evaporation step before spray‐drying, which may have resulted in protein aggregation reducing enzyme digestibility, but more research is needed to confirm this hypothesis.

Based on the calculated DIAAS values, SPHC can be considered a ‘good’ quality protein for children from 6 months to 3 years old and an ‘excellent’ quality protein for individuals older than 3 years, and may be used to complement lower quality proteins.^2^ Viscera were used in the manufacture of SPHC and, despite the fact that viscera are sometimes considered low‐value co‐products, they contain more AA than proteins in heads and bones, indicating that inclusion of viscera in a mixed protein may contribute to improved protein quality.^28^, ^29^ It is therefore likely that it was the presence of viscera in SPHC that resulted in this protein having the greatest DIAAS among the three hydrolysates.

No claims regarding protein quality can be made for SPHI1 and SPHI2 for both age groups, because DIAAS was less than 75 for these two proteins.^2^ However, if a nitrogen factor of 5.60 is used for calculating DIAAS, SPHI2 can be considered as having a ‘good’ protein quality for individuals older than 3 years. Although FAO recommends the use of 6.25 as the nitrogen factor for standardization purposes, ingredients that have a lower nitrogen conversion factor may have their protein quality underestimated if a greater conversion factor is used, because the ingredient AA composition is an important factor used in DIAAS calculations.^2^ A greater DIAAS was also demonstrated for pistachio nuts if a nitrogen conversion factor of 5.30 was used rather than 6.25.^30^

According to published data, the DIAAS in salmon fillet powder used in food preparations is between 86 and 93, having leucine or valine as first limiting AA.^31^ In the present work, leucine or tryptophan was the first limiting AA. Leucine is the AA with the greatest requirements by humans due to its role in muscle protein synthesis;^32^ although the concentration of digestible leucine was relatively high in the hydrolysates, the high requirement for leucine caused this AA to be limiting for both age groups. Tryptophan was also a limiting AA in SPHI1 and SPHI2 for children from 6 months to 3 years old because of its low concentration in salmon hydrolysates and its low SID compared with other indispensable AA, which is common in animal co‐products because of the high concentration of collagen in connective tissue, cartilage, and skin.^33^ Therefore, although both salmon hydrolysate isolates had a high protein concentration, the digestible concentration of leucine and tryptophan was not sufficient to meet requirements for the age groups used in DIAAS calculations. It is likely that the absence of viscera in both protein isolates also contributed to the reduced quality compared with the SPHC.

If the sum of analyzed AA in an ingredient is not close to the CP, it is likely that the protein contains non‐protein nitrogen (NPN). The NPN in salmon hydrolysates may include free nitrates, biogenic amines, and nucleotides due to the presence of bones and cartilage in fish co‐products.^34^, ^35^ The presence of bones may also reduce protein quality because AA in collagen have low digestibility.^33^, ^35^ The SPHC and SPHI1 products contained approximately 7% and 20% NPN, respectively, likely due to the presence of biogenic amines. Indeed, salmon protein hydrolysates had the highest concentration of biogenic amines when compared with other fish co‐products.^19^ Differences in NPN were previously observed among different types of fish co‐products, and were affected by the fish species being processed as well as the raw materials and processing used, with NPN content ranging from 4% to 19%.^19^, ^34^ In porcine and bovine hydrolysates, the NPN content ranged from 1% to 17% and also depended on the processing and raw materials used.^11^

The lower DIAAS values in the SPHI1 product compared with SPHI2 may be due to the high content of NPN and the lower concentration of indispensable AA relative to CP, which is one of the factors that determine DIAAS. The fact that SPHI1 was not ultrafiltrated after separation may have contributed to the increased NPN compared with SPHI2, because NPN compounds are released into the liquid processing streams with most of the water, including potential impurities and larger peptide fragments,^34^ indicating that the ultrafiltration step in the production of SPHI2 enhanced protein quality. This clearly demonstrates that differences in salmon co‐products exist, which may affect their protein quality. It was therefore concluded that the hypothesis that salmon protein hydrolysates can improve protein quality of a meal containing low‐quality proteins can only be partially accepted, because the hypothesis was true for SPHC consumed by individuals older than 3 years, but not if consumed by younger children. For SPHI1 and SPHI2, the hypothesis was rejected because the DIAAS values were too low to allow these proteins to complement other low‐quality proteins.

## CONCLUSIONS

The three salmon hydrolysate proteins used in this experiment have SID values for most AA greater than 85%, indicating excellent digestibility of AA. When compared with FAO requirements for AA, no claims regarding protein quality can be made for SPHI1 because DIAAS was less than 75 for both children and individuals older than 3 years. However, SPHI2 was considered a ‘good’ quality protein for individuals older than 3 years if a lower Jones factor than 6.25 was used, and SPHC was considered a ‘good’ quality protein for children from 6 months to 3 years old and an ‘excellent’ quality protein for individuals older than 3 years. Therefore, SPHC can be used to compensate for ingredients with lower protein quality to produce a diet for individuals older than 3 years that is balanced in all AA.

## AUTHOR CONTRIBUTIONS

All authors conceptualized the research. Hans H Stein supervised the project. Juliana CFR Martins conducted the animal part of the experiment and participated in data interpretation. Natalia S Fanelli analyzed the data and wrote the first draft of the manuscript. Natalia S Fanelli and Hans H Stein revised the manuscript. All authors approved the final version of the manuscript.

## CONFLICT OF INTEREST

The authors have no conflicts of interest.

## Data Availability

The data that support the findings of this study are available on request from the corresponding author. The data are not publicly available due to privacy or ethical restrictions.
